# Phylogenetic Structure and Sequential Dominance of Sub-Lineages of PRRSV Type-2 Lineage 1 in the United States

**DOI:** 10.3390/vaccines9060608

**Published:** 2021-06-05

**Authors:** Igor A. D. Paploski, Nakarin Pamornchainavakul, Dennis N. Makau, Albert Rovira, Cesar A. Corzo, Declan C. Schroeder, Maxim C-J. Cheeran, Andrea Doeschl-Wilson, Rowland R. Kao, Samantha Lycett, Kimberly VanderWaal

**Affiliations:** 1 Department of Veterinary Population Medicine, University of Minnesota, St. Paul, MN 55108, USA; ipaplosk@umn.edu (I.A.D.P.); pamor001@umn.edu (N.P.); dmakau@umn.edu (D.N.M.); rove0010@umn.edu (A.R.); corzo@umn.edu (C.A.C.); dcschroe@umn.edu (D.C.S.); cheeran@umn.edu (M.C.-J.C.); 2 Veterinary Diagnostic Laboratory, University of Minnesota, St. Paul, MN 55108, USA; 3 School of Biological Sciences, University of Reading, Reading RG6 6AS, UK; 4 Roslin Institute, University of Edinburgh, Edinburgh EH25 9RG, UK; andrea.wilson@roslin.ed.ac.uk (A.D.-W.); Rowland.Kao@ed.ac.uk (R.R.K.); samantha.lycett@ed.ac.uk (S.L.)

**Keywords:** porcine reproductive and respiratory syndrome virus, phylogenetic analysis, principal component analyses, mutations, viral population dynamics, whole genome, multi-strain dynamics

## Abstract

The genetic diversity and frequent emergence of novel genetic variants of porcine reproductive and respiratory syndrome virus type-2 (PRRSV) hinders control efforts, yet drivers of macro-evolutionary patterns of PRRSV remain poorly documented. Utilizing a comprehensive database of >20,000 *orf5* sequences, our objective was to classify variants according to the phylogenetic structure of PRRSV co-circulating in the U.S., quantify evolutionary dynamics of sub-lineage emergence, and describe potential antigenic differences among sub-lineages. We subdivided the most prevalent lineage (Lineage 1, accounting for approximately 60% of available sequences) into eight sub-lineages. Bayesian coalescent SkyGrid models were used to estimate each sub-lineage’s effective population size over time. We show that a new sub-lineage emerged every 1 to 4 years and that the time between emergence and peak population size was 4.5 years on average (range: 2–8 years). A pattern of sequential dominance of different sub-lineages was identified, with a new dominant sub-lineage replacing its predecessor approximately every 3 years. Consensus amino acid sequences for each sub-lineage differed in key GP5 sites related to host immunity, suggesting that sub-lineage turnover may be linked to immune-mediated competition. This has important implications for understanding drivers of genetic diversity and emergence of new PRRSV variants in the U.S.

## 1. Introduction

Porcine reproductive and respiratory syndrome virus (PRRSV) is one of the most important pathogens affecting swine globally [1,2,3,4]. In the United States, the annual estimated economic losses due to PRRS are approximately USD 664 million [5], with economic losses stemming from reproductive failure, abortion, premature farrowing, increased rate of stillborn piglets [6], pre-weaning mortality as extreme as 70% among piglets [7], and losses in production parameters such as post-weaning mortality, daily gain, and feed conversion [8,9]. Up to 40% of the U.S. breeding herd experience outbreaks annually [10], which exemplifies the widespread impact of this virus in the U.S.

As demonstrated by the continued endemicity of PRRS in the U.S., efforts to control PRRSV spread have had limited success and are further complicated by the emergence of new genetic variants [11,12]. Current PRRSV vaccines display varying protection against homologous and heterologous challenges [13,14,15,16], and the diversity of wild-type PRRSV variants makes it difficult to predict the nature of immunity elicited by naturally occurring variants against heterologous challenges [17]. Although protection elicited by vaccination or intentional pre-exposure of animals to PRRSV may help mitigate clinical disease impact, current PRRS vaccines and pre-exposure procedures do not produce sterilizing immunity. This may unintentionally create conditions for immune-driven viral adaptation. In addition, recombination between different circulating strains has also been documented [18,19], further illustrating potential risks for viral evolution.

PRRSV is divided into two major viral types—European (type-1) and North-American (type-2) [1,20]. While each of the types is more prevalent on its respective continent, both types can be found across North America, Europe, and Asia [1,20]. PRRSV type-2 viruses are further categorized according to restriction fragment length polymorphisms (RFLP) in the open reading frame 5 gene (*orf5*) portion of the viral genome [21,22]. RFLP typing has recognized shortcomings, which include an inability to represent genetic relationships between different RFLP types, the potential for distantly related viruses to share the same RFLP type [23], and the instability of RFLP types over as few as 10 animal passages [24]. Partially due to these ambiguities in interpreting RFLP types, an alternative classification system based upon phylogenetic lineages was proposed in 2010 [20,25]. This classification system grouped PRRSV type-2 viruses into nine lineages based on phylogenetic relationships in the *orf5* region. The genetic distance between these lineages was approximately 10–17% based on nucleotide identity [25]. Lineages 1, 2, 5, 6, 7, 8, and 9 have been detected in the U.S., with specific lineages more prevalent in certain parts of the country [25]. However these lineages have continued to diversify, and using a dataset from a single U.S. region from years 2009–2017, at least three sub-lineages within Lineage 1 have been documented, with sequential turnover in the dominant lineages through time [23]. In addition, the emergence of new sub-lineages can occur on time scales as short as two years (as observed for the 1A sub-lineage associated with RFLP-type 1-7-4) [23], with rapid spread of emerging sub-lineages driven by animal movements and local area spread [26,27].

Lineage 1 continues to be the most prevalent and diverse lineage within the U.S. swine industry; consequently, an updated and expanded sub-lineage classification system is needed to better track PRRSV Lineage 1 diversity in the U.S. and improve communication and coordination of control efforts. In addition, the macro-evolutionary dynamics of PRRSV circulation in the U.S. remain poorly described at broader and more representative geographic scales, and there is need to advance our understanding of how PRRSV variants emerge and what processes underpin lineage turnover. Additionally, understanding the potential role that host immunity has in shaping viral population dynamics is important, given that PRRSV preventive and mitigation measures in the U.S. often involve the pre-exposure of animals with either modified live vaccines or live virus inoculation. In this paper, we used a comprehensive database of >20,000 *orf5* sequences to investigate phylogenetic sub-structure within Lineage 1 Type-2 PRRSV sequences in the U.S., describe past disease dynamics through quantifying viral population sizes across time, and identify antigenically relevant amino acid changes associated with each sub-lineage.

## 2. Materials and Methods

### 2.1. Source of Sequences

This study was conducted using 21,211 *orf5* sequences from the University of Minnesota Veterinary Diagnostic Laboratory (UMN VDL). The UMN VDL receives samples for diagnostic purposes from throughout the country and is perceived as one of the leading reference VDLs for swine diseases in the U.S. Samples sent for diagnostics usually comprise blood, tissue, or other clinical samples obtained from animals with clinical manifestation compatible with a PRRSV infection, or from farms collecting samples as part of routine monitoring. Diagnosis usually involves an RT-PCR test for the detection of PRRSV and, in selected cases, Sanger sequencing of the *orf5* region of the viral genome—approximately 10% of all PRRSV-positive tests executed at the UMN VDL are sequenced.

At the UMN VDL, multiple samples can be submitted within a single case ID, which usually represent different animals (sometimes pooled) from a single farm. Thus, more than one sequence can be generated for a single case ID. Given that sequences associated with a single farm at a single time point are pseudo-replicates (they more likely represent a single introduction into the farm rather than the introduction of two distinct viruses simultaneously), only the first sequence within each case ID was retained for our analysis in situations where multiple sequences were associated with a single case ID. In addition, sequences for which a complete date of collection was not available were also discarded from the analysis.

### 2.2. Phylogenetic Classification Using Discriminant Analysis of Principal Components

We followed the same rationale for the classification of sequences into lineages as previously published [23]. Briefly, a collection of 841 ORF5 gene sequences served as “anchors” to classify the sequences of this study into one of the nine lineages previously described [20,25]. Sequences were aligned to the anchors using the MUSCLE algorithm in AliView [28]. The aligned data set was imported into Mega 10 [29], where the genetic pairwise distance was calculated as a percentage nucleotide difference. Using Stata 15 [30], each sequence was assigned to the lineage of the most closely related anchor.

**Figure 1:**
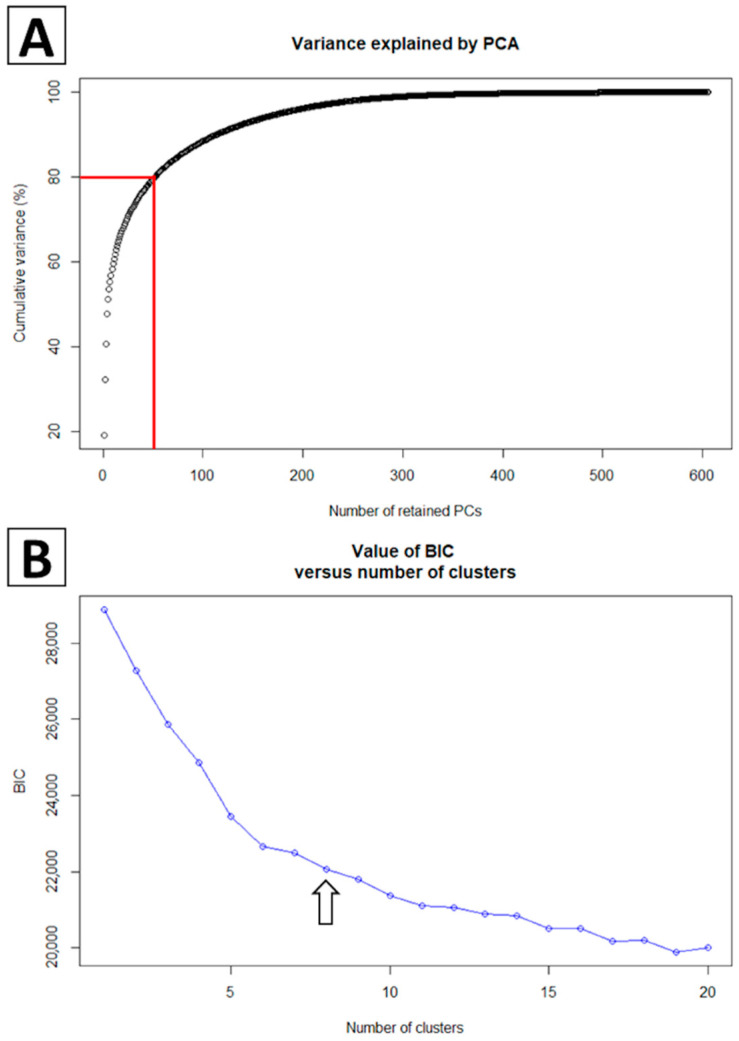
Characteristics of the DPCA used to create sub-lineages within Lineage 1. (**A**) 80% variance was explained when retaining 70 principal components. (**B**) Grouping sequences into eight sub-lineage clusters yielded the lowest BIC while still preserving previously documents sub-lineage structure.

To further refine the classification of lineage 1 sequences, we used a discriminant analysis of principal components (DAPC) available via the package *adegenet* 2.0.0 [31] in R [32]. This method was utilized to uncover the inherent structure present within the Lineage 1 sequence dataset; sequences were clustered such that between-group differences were maximized and within-group variation was minimized. Essentially, this is a principal component analysis, which reduces the dimensionality of the sequence data set into principal components, coupled with a discriminant analysis. We performed this analysis with the unclassified Lineage 1 *orf5* sequences from the UMN VDL alongside 75 Lineage 1 anchor sequences [23]. These anchor sequences, which were previously classified into three sub-lineages (L1A-C), were used as guides to ensure our DAPC protocol was able to adequately reconstruct the previously documented structure. We retained 70 principal components, accounting for 80% of the genetic data variance, in the discriminant analysis (Figure 1A). The optimal number of clusters in which to group sequences was defined as the number of clusters that yielded the smallest Bayesian information criterion (BIC) in the DAPC analysis while preserving the previously described sub-lineage structure.

The rate at which recombinant sequences were present in our data set was determined by detecting recombinants on a subset of data (due to limited bioinformatics available to handle a data set as extensive as ours), and we found that the frequency of *orf5* recombinants was ~0.1% (which would amount to fewer than 20 recombinants in all our sequences). The identification of recombinants in the entire data set was precluded due to inherent limitations of bioinformatics for data sets this large. Although recombinants may be present in the dataset, the random sub-sampling of sequences for further analysis diminishes the impact they may have had, since the likelihood that recombinants were present in sub-sampled runs of the further analyses is reduced.

### 2.3. Effective Population Size through Time

The temporal signal in phylogenetic data sets of each sub-lineage was first investigated using TempEst to confirm the appropriateness of the data for time-scaled phylogenetic tree reconstruction [33]. We then used Bayesian SkyGrid coalescent models to estimate past population dynamics through time [34]. Briefly, this approach estimates a sub-lineage’s effective population size by evaluating the genetic diversity through time under an idealized reproductive model [34]. In comparison to raw sequence counts, this approach is less sensitive to undersampling (such as reduced sequence availability in earlier years), and oversampling where many highly-similar sequences are obtained (such as in an outbreak investigation), thus improving our ability to discern population-wide lineage turnover through time. A relaxed uncorrelated lognormal (UCLN) molecular clock was used, with a flexible Bayesian SkyGrid plot (BSP) demographic model and a general-time reversible model of nucleotide substitution with gamma-distributed rate variation among sites (GTR+Γ), allowing for partitions into codons in any of three positions [35]. Models were run with 200 million Markov chain Monte Carlo repetitions per run, sampling one of each 1000 trees, which was sufficient to obtain a stable posterior distribution. These analyses were implemented using BEAST (v1.10.4) on XSEDE on the CIPRES Cyberinfrastructure for Phylogenetic Research [36]. The steps above were replicated three times for each PRRSV type-2 lineage and sub-lineage found in the data. Due to computational constraints, each replicate analysis was performed on a different set of 300 sequences of each lineage/sub-lineage that were randomly selected from the total pool of sequences of each lineage/sub-lineage. BEAST results of the three runs belonging to the same lineage/sub-lineage were combined using LogCombiner [37]. The individual and combined BEAST results were read into Tracer to evaluate model convergence and consistency between replicates; the individual and combined model outputs were used to reconstruct SkyGrid plots of the estimated viral population size through time [38]. Overall, individual and combined BEAST runs of the same lineage/sub-lineage yielded similar results, and estimated viral population sizes across time were consistent. Results were exported and plotted using Stata [30].

Each sub-lineage’s year of emergence and year in which it reached peak population size were determined based on the SkyGrid effective population size results. The year of emergence was defined as the first of consecutive years in which the estimated viral population size increased by a factor of two or more as compared to the previous year. Although a given sub-lineage may sometimes be detected much earlier than this date, this definition of emergence corresponded to periods of time in which rapid expansion in effective population sizes were visibly evident in SkyGrid plots, likely reflecting widespread transmission of the sub-lineage. If such population expansions were observed in two non-consecutive points of time, then both were flagged as (re-)emergences. A sub-lineage’s peak was defined as the year with the highest estimated viral population size, or for sub-lineages with several emergence and re-emergence events, the year with the highest estimated viral population size between the two emergence events.

### 2.4. Clade-Prevalent Mutations

In order to better understand the significance of genetic differences between sub-lineages, we extracted consensus amino acid sequences for each sub-lineage, with the threshold of consensus set to 50%, 75%, and 90% of sequences within a sub-lineage possessing a particular amino acid at a particular site. Consensus *orf5* sequences were assembled using Geneious Prime^®^ [39]. The *orf5* sequences of five commercially available vaccines in the U.S. (Ingelvac PRRSV ATP–GenBank ID DQ988080.1, Ingelvac PRRSV MLV–GenBank ID AF066183.4 (both from Boehringer Ingelheim), Fostera PRRSV–GenBank ID KP300938.1 from Zoetis, Prime Pac PRRSV RR–GenBank ID DQ779791.1 from Merck, and Prevacent GenBank ID KU131568.1 from Elanco) were added to aid in comparisons between these vaccines and the consensus sequence of each sub-lineage.

### 2.5. Comparison of Databases: UMN VDL & GenBank

To evaluate how representative the UMN VDL database is of the PRRSV type-2 diversity in the U.S., we downloaded all type-2 *orf5* PRRSV sequences from GenBank submitted from the U.S. and Canada up to 21 December 2020. GenBank sequences were categorized as those that were available when the lineage classification of type-2 PRRS was originally proposed (an analysis that included all available Genbank sequences up to Jan 2009) [25], sequences contributed by our group in a prior publication [23], and other GenBank sequences. GenBank sequences were classified into lineages/sub-lineages using a set of anchors selected from each linage (L2–L9) and sub-lineage within L1 (Lineage 1A–1H). All sequences (GenBank and UMN VDL) were aligned using MAFFT [40]. A maximum likelihood tree was built using the GTR model using IQ-TREE [41] and a time-scaled tree was inferred using TreeTime [42] under a strict clock model. A phylogenetic tree with the combined UMN VDL/GenBank data set was then illustrated using Nextstrain 2.0.0 [43].

## 3. Results

**Figure 2:**
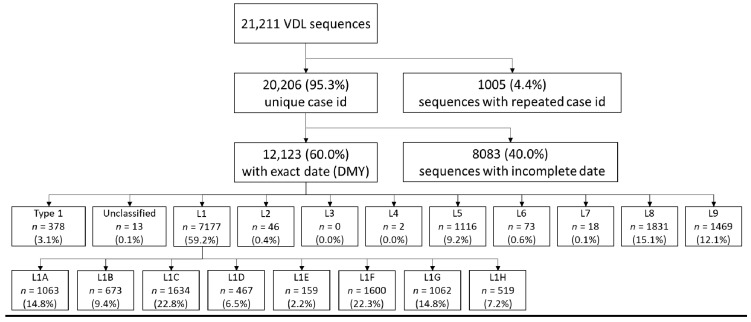
Flowchart describing the number of PRRSV sequences classified into each lineage and sub-lineage L1 after removing duplicates and sequences without a date.

We obtained 21,211 *orf5* PRRSV sequences from the UMN VDL representing samples from 2001–2018. A total of 1005 duplicated sequences originating from the same case IDs were excluded. Among the 20,206 unique case ID sequences, dates were not available for 8083 sequences. Therefore, 12,123 sequences were carried over to the lineage/sub-lineage assignment protocol. For comparison, by mid-February 2021, GenBank contained approximately 26,500 PRRSV *orf5* sequences from throughout the world (including both type-1 and type-2 PRRSV). Approximately 59% of our sequences were classified as Lineage 1. Lineage 8 was the second most prevalent lineage, accounting for approximately 15% of our sequences. The number of sequences classified in each lineage can be found in Figure 2.

**Figure 3:**
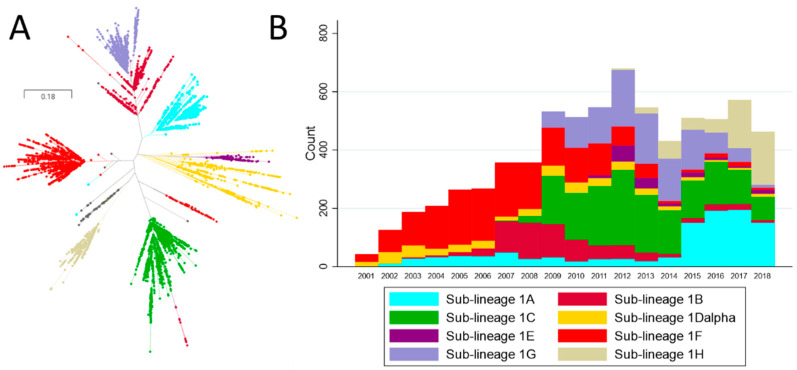
(**A**) Phylogenetic tree of L1 sequences classified into sub-lineages according to discriminant analysis of principle components. The gray tips in the tree represent sub-lineage 1D-beta sequences, which are not displayed in other analysis. (**B**) Absolute frequency of sequences per sub-lineage per year.

Sequences belonging to Lineage 1 were further stratified into sub-lineages using DAPC, which yielded 8 genetic groups within Lineage 1 denoted as sub-lineages 1A–1H. An extensive set of reference sequences from the UMN VDL belonging to each lineage and sub-lineage can be found on GenBank (accession numbers MZ303973–MZ304662). The mean genetic distance within and between sub-lineages is shown on Table 1 (amino acid distances are shown in Table S3). Overall, genetic distance between sub-lineages was typically 13–15%, with the exception of the genetic distance between sub-lineage 1B and 1G, which was approximately 6% (lineage 1G appeared to have emerged from 1B; Figure 3A). The average genetic distance within sub-lineages was <6%, with the exception of sub-lineage 1D (Table 1). Whereas most sub-lineages formed well-resolved phylogenetic clades (Figure 3A), sub-lineage 1D was a poorly resolved group containing sequences belonging to two distinct clades, in addition to several poorly classified sequences (Figure 3A). Attempts to increase the number of groups formed by DAPC to further subdivide this sub-lineage resulted in misclassification of previously described 1A, 1B, and 1C sequences. Therefore, the largest clade within 1D was denoted as 1D-alpha. The remaining 1D sequences were denoted as 1D-beta. 1D-beta was a particularly poorly resolved group containing the smaller 1D clade and other 1D sequences not clearly belonging to any clade. Due to these issues, sub-lineage 1D-beta was removed from further analysis.

RFLP types of all sequences were obtained, and the frequency of RFLP types within each sub-lineage can be found in Table S1, although we caution that sub-lineages and RFLPs are not simply different naming schemes capturing similar sub-structure within PRRSV type-2. Overall, the most frequent RFLP type identified in the UMN VDL dataset was 1-8-4, accounting for approximately 24% of the UMN VDL sequences. Approximately 73% of the 1-8-4 sequences belonged to sub-lineage 1F (mostly years 2004–2012), whereas newer 1-8-4 sequences (mostly between 2013–2018) belonged to sub-lineage 1H. Within a sub-lineage, RFLP frequency varied through time. For example, overall, the most frequent RFLP type within sub-lineage 1A was 1-7-4 (approximately 60% of all 1A sequences), yet 1-7-4 frequency was 20% between 2001–2013 and 80% between 2014–2018.

Sub-lineage 1C was the most prevalent sub-lineage identified overall, representing approximately 22.8% of all Lineage 1 sequences (Figure 2). However, sub-lineage prevalence did not remain constant through time (Figure 3B), and periods in which specific sub-lineages were more prevalent are clear. Sub-lineage 1C, for example, first appeared in our data set in 2007 and was responsible for roughly 30–50% of all sequences identified in any given year from 2010–2014. Sub-lineage 1F, on the other hand, was most prevalent between circa 2002 until 2008, and has rarely been detected since 2014. Of note is the sudden increase in the detection of certain sub-lineages, such as sub-lineage 1A. This group was consistently detected across the early years of our data, but beginning in 2015, it displayed a marked increase in its occurrence, and subsequently has been responsible for ~30 to 40% of all detected sequences.

### 3.1. Sequential Dominance of Sub-Lineages through Time

**Figure 4:**
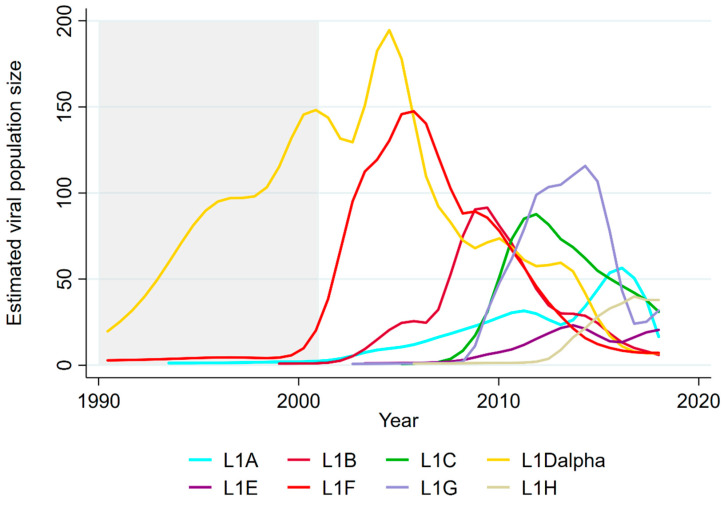
Estimated viral population through time for each L1 sub-lineage. The area in gray denotes the period in which no sequences were available.

Because sequencing effort has changed through time, the absolute frequency of sequences is challenging to interpret. Therefore, we quantified the expansion and contraction of each sub-lineage’s effective population size using a SkyGrid analysis (Figure 4), which was based on reconstruction of population trends based on genetic diversity in time-scaled trees and was less sensitive to sampling effort. All sub-lineages displayed a sufficiently strong temporal signal (correlation between genetic divergence and sampling time, r^2^, for different sub-lineages ranged between 0.18 and 0.67) to be suitable for phylogenetic analyses involving molecular clocks. The estimated viral substitution rates were similar across sub-lineages, ranging from 6.6 × 10^−3^ to 1.3 × 10^−2^ substitutions/site/year (Table S2). Figure 4 shows the SkyGrid plots of each sub-lineage overlaid to facilitate comparisons of the effective population size of each sub-lineage through time; SkyGrid plots with 95% HPD intervals are shown individually in Figure S1. Table 2 shows the year of (re-)emergence and peak of each sub-lineage. On average, we observed the emergence or re-emergence of a sub-lineage approximately every 1–4 years, and the time between sub-lineage emergence and its peak estimated viral population size was approximately 4.5 years on average. Two sub-lineages had more than one emergence and peak (L1A and L1E).

This analysis demonstrated a clear turnover in the dominant sub-lineage (sub-lineage with the largest population size) across years, with a different dominant sub-lineage peaking every ~3 years (Table 2). The sub-lineage within Lineage 1 with the earliest date of emergence was L1D-alpha, which was estimated to have emerged prior to 1990 and reached its peak in 2004, though the sparsity of older sequences led to greater uncertainty about the early dynamics of this sub-lineage. Prior to L1D, SkyGrid analyses of non-Lineage 1 PRRSV revealed that Lineage 9 peaked in 2001 and Lineage 5 peaked in 1998, which was a historical extension of the three-year cycle of lineage turnover observed for the sub-lineages within Lineage 1 from 2004 to 2018. In the last three years of data (2015–2018), the sub-lineage with the highest estimated viral population size was L1A, which peaked in 2016, although L1H had the largest population size in 2018. A post hoc analysis of additional sequences from 2019–2020 revealed that the relative frequency of L1H and L1A in VDL *orf5* sequences from 2019 and 2020 remained relatively stable, with ~25% of sequences in 2019 and ~37% in 2020 belonging to L1H, and ~45% and ~38% belonging to L1A.

### 3.2. Clade-Prevalent Mutations

**Figure 5:**
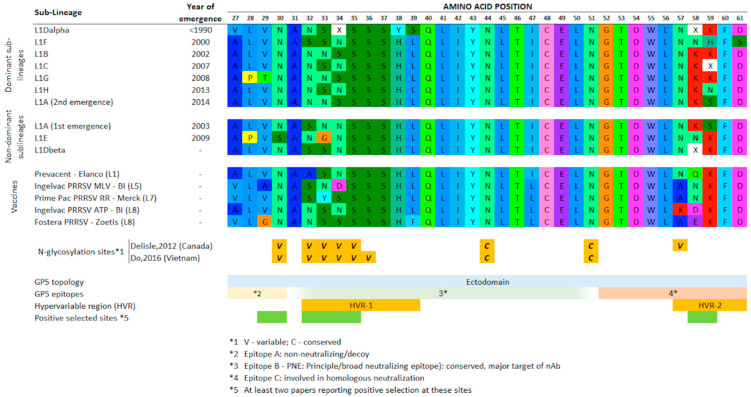
Consensus amino acid sequences of the ectodomain portion of GP5 (AA positions 27–61) for each sub-lineage, wherein the amino acid is present in >50% of sequences in a given sub-lineage. Sub-lineages in this figure are ordered according to their time of emergence to give a better idea of the immune landscape immediately prior to each sub-lineage’s emergence. *5: papers considered include [23,44,45,46,47].

The 50% consensus amino acids sequences of each sub-lineage’s ectodomains (amino acid positions 27–61) are shown in Figure 5, and the 75% and 95% consensus sequences for the whole ORF5 are shown in Supplementary Figure 2. Fewer amino acids reached the consensus level as the threshold was increased; thus, the 75% and 95% consensus sequences revealed highly conserved sites, whereas the 50% consensus revealed substitution patterns that differentiated sub-lineages for the majority of sequences (Supplementary Figure S2). Within the hypervariable regions, for example, we observed many differences in the amino acids coded for at N-glycosylation sites (sites 32–34) or neutralizing epitopes (mainly in sites 58–59). When the dominant sub-lineages were ordered by the year of emergence to approximate population immunity potentially present immediately prior to each sub-lineage’s emergence (Figure 5), there was no instance in which consecutively emerging sub-lineages shared the same consensus pattern in positions 32–34 and 58–59 (although in the latter case, the consensuses at 50% prevalence within each sub-lineage were not always defined).

## 4. Discussion

Here, we delineate sub-lineage structure and macro-evolutionary dynamics within PRRSV Lineage 1 detected in the U.S. from 2001 to 2018. By applying a genetic clustering algorithm to more than 7000 *orf5* PRRSV Lineage 1 sequences, we found that the best delineation of genetic diversity with Lineage 1 was achieved by grouping sequences into eight sub-lineages. While whole genome sequencing (WGS) data would unveil a broader perspective on PRRSV evolution, recent data has shown that sequences grouped together as sub-lineages in *orf5* phylogenies largely remain grouped by WGS [48], suggesting that *orf5*-based lineage classification does reveal groups of viruses with shared ancestry. Here, we show that each sub-lineage emerged and circulated in different periods through time, showing a pattern of sequential turnover in the dominant sub-lineage. PRRSV is characterized by a rapid rate of evolution, which represents a major obstacle for its control [49], since genetic changes may affect virus neutralization [50,51]. An examination of common amino acids within each sequential sub-lineage revealed differences at key amino acid sites (sites 33–34) that others have shown to be under positive selection pressure [23,45,46], and have been shown experimentally to be linked to immune escape [52]. While the observational nature of our data makes causality of sub-lineage emergence difficult to assess, the findings of this study support hypothesis generation on processes that lead to the observed turnover in sub-lineages through time. Taken together, our results support the idea that PRRSV evolution and continued endemicity in the U.S. is characterized by multi-strain dynamics driven at least partly by immune-mediated interactions [23,53].

While antigenic differences and the extent of immunological cross-protection among sub-lineages described here has not been directly assessed, *orf5* encodes for the major envelope glycoprotein (GP5) that plays a key role in inducing virus neutralizing antibodies, and particular amino acids encoded by ORF5 mediate the viruses susceptibility to neutralization by the immune system [49,50,54,55]. Our results suggest that antigenic differences may be captured to some extent by phylogenetic classification based on *orf5*, though we acknowledge that whole-genome sequence data would likely reveal additional antigenic differences in genomic regions outside of *orf5* that play a role in immunity [49]. Within *orf5*, amino acid differences between sub-lineages (Figure 5) occur at sites that are known to be involved with viral recognition or with immune response by the swine host, such as hypervariable regions 1 and 2 (spanning between amino acid positions 32–39 and 57–61 [56,57,58]). Research suggests that these hypervariable regions evolve under positive selection pressure [23,44,45,46] and contain several variable N-glycosylated sites [45,46] that play an important role in host immune evasion [52,59,60]. However, differences in the amino acids in other portions of the GP5 protein were also present; e.g., the unique amino acids valine, threonine, and serine in the non-neutralizing epitope (amino acid positions 27–30) [61], which were more prevalent in L1D-alpha, L1G, and L1E, respectively. These results are important because they lend greater strength to the hypothesis that viral infections by different sub-lineages within Lineage 1 may yield different immunological responses in the animal, which could contribute to co-circulation of multiple PRRSV strains and the antigenic fitness of newly emerging variants in a given region, system, or farm.

The alternating pattern of the presence of N-glycosylation at sites 33–34 across sequentially emerging sub-lineages suggests that viruses that are antigenically distinct from the previous dominant sub-lineage may have a fitness advantage [45,46], and thus are able to successfully emerge against an immune backdrop shaped by its predecessor. While whole-genome sequence data is needed to resolve this picture, our observations suggests that there may be immunological differences between the ORF5-based lineage/sub-lineage that may help explain the emergence/re-emergence of PRRSV lineages identified in the U.S. Whole-genome sequencing data combined with further investigations examining how point mutations affect protein secondary structure, protein folding, and post-translational modifications may further help clarify the potential of such mutations in altering virus recognition by the host immunological system.

To overcome limitations in inferring temporal patterns from raw numbers of sequences identified through time, we described past viral population dynamics using a Bayesian coalescent analysis that allowed us to better refine years of emergence and peak of each sub-lineage, even permitting for the estimation of the average number of years that elapsed between each sub-lineage’s emergence and peak. This approach allows us to minimize potential biases in estimating the viral population size that would emerge if based purely on raw frequencies. For example, if an extensive PRRSV outbreak investigation results in the generation of many sequences, the inclusion of numerous closely related sequences contributes relatively little additional genetic diversity to the data set, and thus has a minimal impact on estimation of effective population sizes. Similarly, effective population size estimates are robust to the sparsity of sequence data early in the study period, as long as sampling was reasonably representative.

**Figure 6:**
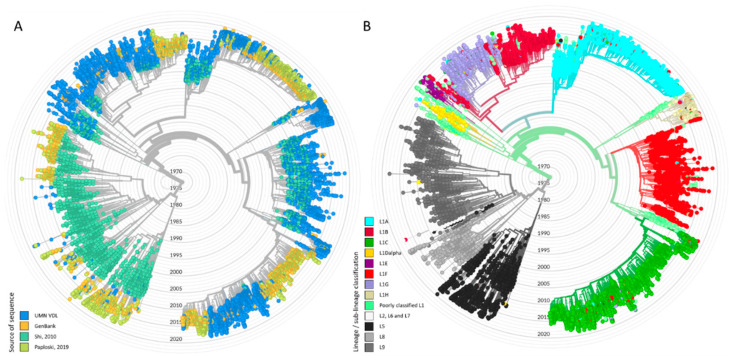
(**A**) Nextstrain tree illustrating sequences from the UMN VDL and from GenBank according to its source, and (**B**) according to its lineage/sub-lineage.

Systematic biases in sampling could result in some phylogenetic clades being undetected. In order to evaluate the representativeness of the UMN VDL database, we obtained all type-2 *orf5* PRRSV sequences from GenBank submitted from the U.S. and Canada, and built a phylogenetic tree with the combined UMN VDL/GenBank dataset using Nextstrain (Figure 6) [43]. All major clades found in GenBank were also found in the UMN VDL data, particularly within Lineage 1. Thus, we believe that the sub-lineages within Lineage 1 described here are representative of the current diversity within Lineage 1 present in the U.S. While the UMN VDL does provide sequencing services for clients across the U.S., its location in the Midwest corn belt makes it particularly useful as a source of data for PRRSV diversity, given that large numbers of pigs from throughout the U.S. and Canada are moved into this region for finishing, and thus viruses circulating elsewhere in the U.S. are very likely to be transported into the Midwest [25]. That being said, spatial heterogeneities in the occurrence of sub-lineages likely exist, given that intensive swine production is concentrated in certain parts of the country. This is apparent from our prior work, in which we only identified three sub-lineages within a single U.S. production region between 2009 and 2017 [23], while this paper demonstrates that additional distinct sub-lineages circulated in other parts of the country. It is also important to note that the proposed sub-lineages were based on data from the U.S. only, and thus reflect phylogenetic structure present within the U.S. and perhaps Canada (due to the connectivity of the two countries’ swine industry), and are not meant to encompass diversity on other continents. Sub-lineages (and past population dynamics) found on other continents are expected to be distinct from those documented here [62,63,64].

We identified that sub-lineages emerge and peak over time (Figure 4 and Table 2), with an average of 4.5 (ranging from 2 to 8) years elapsing between the emergence and peak, followed by a decline of the estimated viral population size of a sub-lineage. This pattern was true for all sub-lineages except L1D-alpha (early dynamics were difficult to quantify, as its period of emergence preceded sequence availability; Table 1) and L1F. The consistency of this pattern for most sub-lineages within Lineage 1 suggests that there may be something intrinsic to the U.S. swine industry structure that may define the upper limits of viral population size, possibly determined by dynamics of spread within and between different swine-producing regions of the U.S.

Only two sub-lineages had a secondary re-emergence event following initial peaks (Lineage 1A and 1E). From the raw frequencies, it is apparent that L1A’s second emergence in 2014 was rapid, widespread, and more significant that its original emergence, whereas L1E is of less interest, as it never became a dominant sub-lineage. L1A thus warrants further discussion, since the two distinct emergence events represent separate clades, further reinforcing the hypothesis that sub-variants within sub-lineages exist. Stratifying L1A sequences into early and late clades, there were distinctions between the consensus sequences in both the ectodomain (positions 32 and 34; Figure 5) and at position 121 (Figure S2). These clades emerged in different moments in time, at which the immunological landscape was likely different. The first emergence of L1A occurred circa 2003, when the most prevalent sub-lineage within Lineage 1 was L1D-alpha, followed by L1F and when Lineage 9 sequences were also circulating more intensely (data not shown); while the second emergence occurred in 2014, after a very complex turnover of sub-lineages within Lineage 1 had occurred in the previous years (Figure 4), and shortly after a drastic increase in the use of a modified live vaccine (belonging to Lineage 5) across the industry. It is possible that the different immunological landscapes shaped evolutionary pressures such that it favored the “re-emergence” of a slightly different L1A clade. Variants within other sub-lineages may also have distinct immunological profiles that allow them to re-circulate despite being of the same sub-lineage, thus population immune dynamics among variants likely occur within as well as between sub-lineages, as exemplified by L1A. The distinction between L1A’s two emergence events is also captured by examining the RFLP-types associated with each clade: from 2001 to 2013, 49.5% and 19.0% of the L1A sequences were classified as RFLP types 1-4-4 and 1-7-4, respectively. Between 2014 and 2018, this frequency changed to 6.3% and 78.9%, respectively. While it is tempting to draw direct parallels between sub-lineages and RFLP types, the relationship between both is more complex than simply a different naming scheme. Thus, the exact frequency of RFLP within each sub-lineage can change depending on when the data being referred to was collected.

Our paper further supports previous studies [23,25,65] that the phylogenetic classification of PRRSV using *orf5* provides more accurate evolutionary insights into the genetic diversity of PRRSV than the current industry standard of RFLP-typing. Although phylogenetic classification may not discriminate among closely related variants, lineage/sub-lineage classification minimizes ambiguities that occur when typing PRRSV using RFLP; for example, the potential for distantly related virus (e.g., of different lineages) to have the same RFLP pattern. It also diminishes the issue originating from the intrinsic instability associated with RFLP typing, which has been shown to yield different RFLP patterns of a PRRSV virus in as few as 10 animal passages [24]. While sub-lineages may not have sufficient resolution to be used as sole definition for localized outbreaks [66], they may be helpful in avoiding RFLP-based case definitions that group together viruses that are not phylogenetically related, as well as in elucidating the potential evolutionary origin of novel variants. This may help reduce noise when conducting outbreak investigations and perhaps provide more meaningful answers to the industry.

Further investigation into how well sub-lineages defined by using *orf5* carry over to phylogenies based on whole-genome sequencing is warranted. While sequences grouped in the same sub-lineages defined here largely remain grouped together in the same clades in WGS [48], it is less clear whether ancestral relationships between sub-lineages will be preserved in all cases. In addition, specific mutations and viral subpopulations may only be detectable using whole-genome sequencing [67]. However, adoption of whole-genome sequencing in the industry must become routine for this information to be of practical use, and there is concern that WGS is more likely to be preferentially performed for selected viruses, based on (for example) unusual clinical presentation. The historical availability of *orf5* sequences must not be understated, and such large-scale data sets provide unique opportunities to understand the natural history of the virus, despite being intrinsically limited to *orf5*. Epidemiological and evolutionary studies that aim to reconstruct historical trends and past epidemic patterns based upon available historical sequences are likely to be restricted to *orf5*, at least for now, simply due to practical limitations in obtaining equivalent whole-genome data. Still, other regions of the genome are likely to harbor important immunological sites and provide useful additional insights into PRRSV phylogeny macro-evolution, and that important immunological sites may be present elsewhere other than orf5 remains.

Besides lacking a whole-genome perspective, a limitation of our amino acid consensus approach is that it glosses over geographical or temporal variation within a given sub-lineage, and as such, we may be underestimating differences between variants. Despite that, we were still able to detect differences in immunologically relevant sites, and the sub-lineage classification here at least partially captures potential antigenic substructure that seems to exist within Lineage 1. Another limitation of our work is the proportion of sequences that were unable to be used on our analysis (40%, Table 1) due to the inability to associate a date with the sequence. This high proportion of losses may have potentially introduced biases if the distribution of these loses in time is not random. However, we believe that this loss was random, since it originated mostly from lack of information at sample submission.

Last, studies investigating variability in cross-neutralization among sub-lineages would shed light on processes driving sub-lineage emergence and multi-strain dynamics documented herein, and may provide important insight into selection of appropriate vaccines or inoculums that provide the best immunological protection. Such studies will provide important insights into viral evolution, emergence of new immune-escape variants, and how to better immunize animals in order to minimize infection and maximize production of healthy animals.

## 5. Conclusions

Here, we described the occurrence of PRRSV over 17 years in the U.S. using data from one of the largest veterinary diagnostic laboratories in the country. We identified the emergence and turnover of different lineages and sub-lineages in the commercial pig population based on both sequence count data and estimated past viral population sizes inferred from genetic diversity through time. The eight sub-lineages identified within Lineage 1 differed in key amino acid sites of the GP5 that are thought to be involved in the immune response to the virus. This further lends strength to the hypothesis that immune-mediated competition or selection may drive the emergence of new sub-lineages within Lineage 1 in the U.S. Additionally, the interactions in the immune response elicited against these different sub-lineages warrants further investigation to provide insights into herd protection via vaccination, disease control, and viral evolution.

## Figures and Tables

**Table 1:** Genetic distance (% nucleotide difference) within and between lineages and sub-lineages within Lineage 1 defined using the DPCA (including anchor sequences used as reference for sub-lineages 1A, 1B, and 1C). Light gray cells show the average genetic distance of all sequences in each lineage and sub-lineage to commercially available PRRS vaccines.

Lineage	L1A	L1B	L1C	L1D	L1Dalpha	L1Dbeta	L1E	L1F	L1G	L1H	L2	L4	L5	L6	L7	L8	L9
	n = 1095	n = 683	n = 1663	n = 469	n = 405	n = 64	n = 161	n = 1600	n = 1062	n = 519	n = 46	n = 2	n = 1116	n = 73	n = 18	n = 1831	n = 1469
**L1A**	4.0																
**L1B**	10.1	4.0															
**L1C**	13.4	14.5	6.0														
**L1D**	12.6	14.4	14.2	12.0													
**L1Dalpha**	12.7	14.6	14.7	12.0	12.0												
**L1Dbeta**	11.8	13.6	11.4	12.2	13.0	7.0											
**L1E**	14.9	15.7	16.2	14.6	14.6	14.6	5.0										
**L1F**	11.7	13.7	12.5	13.6	13.9	11.4	15.5	6.0									
**L1G**	11.0	6.2	15.0	15.2	15.4	14.0	16.3	14.0	5.0								
**L1H**	14.2	15.9	14.7	14.7	15.3	11.4	17.1	13.1	15.9	5.0							
**L2**	15.4	16.6	17.5	15.4	15.5	15.4	16.3	16.2	17.2	16.8	12.0						
**L4**	14.9	15.6	16.7	15.3	15.3	15.3	17.1	15.8	16.7	17.3	16.6	16.0					
**L5**	14.2	15.4	16.9	14.0	13.9	15.0	16.7	16.4	16.0	17.7	13.5	14.7	4.0				
**L6**	17.3	18.5	19.4	16.6	16.5	17.5	18.8	18.1	18.9	17.8	17.0	17.4	13.4	6.0			
**L7**	13.8	16.0	16.0	13.6	13.5	14.1	15.8	15.8	17.0	16.9	14.2	14.8	11.3	14.7	5.0		
**L8**	15.3	16.0	17.1	14.2	14.0	15.3	16.4	16.6	17.2	17.5	14.7	15.7	12.0	15.1	12.4	6.0	
**L9**	16.8	18.0	17.9	15.0	14.9	15.7	17.7	17.3	18.7	17.7	15.8	16.9	13.7	15.0	12.2	12.5	11.0
**Prevacent-Elanco (L1)**	12.4	15.0	12.8	13.2	14.1	7.5	15.7	12.5	15.1	12.2	16.8	16.6	15.7	19.0	14.5	16.9	16.8
**Ingelvac PRRSV MLV-BI (L5)**	13.9	15.3	16.8	13.7	13.5	14.8	16.7	16.1	15.8	17.6	13.2	14.3	2.2	12.7	10.8	11.7	13.5
**Prime Pac PRRSV RR-Merck (L7)**	13.8	16.0	16.1	13.3	13.2	13.9	16.5	15.7	17.0	16.5	13.8	14.7	10.6	14.1	3.2	11.7	11.4
**Ingelvac PRRSV ATP-BI (L8)**	14.8	15.4	16.9	13.8	13.6	15.2	15.9	16.3	16.7	17.4	14.3	15.2	11.2	14.8	12.3	4.0	12.4
**Fostera PRRSV-Zoetis (L8)**	14.6	15.9	16.2	12.6	12.4	14.3	15.4	15.9	16.7	17.1	13.0	14.1	9.8	12.4	8.8	7.3	9.3

**Table 2:** Year of emergence and peak of different sub-lineages. Sequences are ordered according to year of emergence. Cells highlighted in gray mark sub-lineages that were the most prevalent in the population in the year of its peak. * Ongoing, as our sequence data only extends to 2018.

Sublineage	Year of 1st Emergence			Year of 2nd Emergence		
	Emergence	Peak	Difference between Emergence and Peak	Emergence	Peak	Difference btween Emergence and Peak
L1Dalpha	<1990	2004	>15	-	-	-
L1F	2000	2005	5	-	-	-
L1B	2002	2008	6	-	-	-
L1A	2003	2011	8	2014	2016	2
L1C	2007	2011	4	-	-	-
L1G	2008	2014	6	-	-	-
L1E	2009	2014	5	2016	2018 *	2
L1H	2013	2017 *	4	-	-	-

## Data Availability

The data that support the findings of this study may be available upon reasonable request to the corresponding author (K.V.). The data are not publicly available, as they are part of diagnostic data from third parties (companies and veterinarians submitting samples for diagnosis to the UMN VDL).

## Supplementary Materials

The following are available online at https://www.mdpi.com/article/10.3390/vaccines9060608/s1, Figure S1. Estimated population size (95% HPD) through time of the combined runs of BEAST for each sub-lineage. Figure S2. Consensus amino acid for each position in the ORF5 genome of the different sub-lineages within L1, according to prevalence within that sub-lineage (50, 75, and 95%, respectively). Table S1. Absolute frequency of different RFLP types in each sub-lineage within Lineage 1, sorted according to the most frequent RFLP found in the entire dataset. Table S2. Substitution rate found on the different BEAST runs of samples for each sub-lineage. Table S3. Percent amino acid difference between and within lineages and sub-lineages within Lineage 1. Light gray cells report the average amino acid distance of all sequences in each lineage and sub-lineage to commercially available PRRS vaccines.
