# Supplementary material for: Phylogenetic Structure and Sequential Dominance of Sub-Lineages of PRRSV Type-2 Lineage 1 in the United States
Source: Vaccines (Basel). 2021 Jun 5;9(6):608. doi: 10.3390/vaccines9060608 (PMC8229766; doi:10.3390/vaccines9060608)
Supplement: Supplementary file 1 [file vaccines-09-00608-s001.zip › Supplementary Tables Paploski VDL.pdf]

Table S1

	Sub-lineage								
**RFLP**	**1A**	**1B**	**1C**	**1Dalfa**	**1Dbeta**	**1E**	**1F**	**1G**	**1H**
1-8-4	4	0	7	7	30	0	1225	0	416
1-4-4	211	0	1063	63	0	0	51	0	44
1-26-2	0	12	0	0	0	0	0	683	0
1-7-4	640	0	11	0	0	0	0	0	0
1-18-2	0	428	0	0	0	0	0	156	0
1-3-4	3	0	257	4	4	7	2	0	3
1-4-2	14	0	44	93	1	1	33	0	1
1-4-3	9	0	97	19	0	0	1	0	3
1-3-2	0	0	21	19	0	76	5	6	0
1-18-4	0	113	0	0	0	0	0	0	0
1-2-4	3	15	70	5	0	0	18	0	1
1-16-4	0	0	0	0	1	0	104	0	1
1-8-2	0	0	0	77	3	0	18	0	6
1-37-2	0	1	0	0	0	0	0	102	0
1-8-3	0	0	3	18	7	0	35	0	13
1-12-4	0	0	1	0	4	0	48	0	17
1-22-2	0	0	0	0	0	61	0	0	0
1-7-2	54	0	0	5	0	0	0	0	0
1-13-2	7	13	4	0	0	0	3	14	0
1-26-4	0	10	0	0	0	0	0	22	0
1-2-2	7	11	1	7	2	0	1	3	0
1-7-3	31	0	1	0	0	0	0	0	0
2-5-2	0	0	0	26	0	0	0	0	0
Others	80	70	54	61	11	14	56	76	14

Table S2

Sub-lineage	Run	Substitution Rate
L1A	combined	7.72E-03
L1A	1	7.62E-03
L1A	2	7.89E-03
L1A	3	7.63E-03
L1B	combined	8.77E-03
L1B	1	8.49E-03
L1B	2	9.05E-03
L1B	3	8.76E-03
L1C	combined	1.22E-02
L1C	1	1.17E-02
L1C	2	1.23E-02
L1C	3	1.25E-02
L1Dalpha	combined	8.38E-03
L1Dalpha	1	8.48E-03
L1Dalpha	2	8.74E-03
L1Dalpha	3	7.91E-03
L1Dbeta	combined	NA
L1Dbeta	1	6.65E-03
L1Dbeta	2	NA
L1Dbeta	3	NA

Sub-lineage	Run	Substitution Rate
L1E	combined	NA
L1E	1	8.47E-03
L1E	2	NA
L1E	3	NA
L1F	combined	6.95E-03
L1F	1	7.48E-03
L1F	2	7.06E-03
L1F	3	6.47E-03
L1G	combined	1.09E-02
L1G	1	1.02E-02
L1G	2	1.12E-02
L1G	3	1.11E-02
L1H	combined	9.37E-03
L1H	1	9.08E-03
L1H	2	9.00E-03
L1H	3	9.98E-03

Table S3

Lineage	L1A	L1B	L1C	L1D	L1Dalpha	L1Dbeta	L1E	L1F	L1G	L1H	L2	L4	L5	L6	L7	L8	L9
	n = 1095	*n* = 683	*n* = 1663	*n* = 469	*n* = 405	*n* = 64	*n* = 161	*n* = 1600	*n* = 1062	*n* = 519	*n* = 46	*n* = 2	*n* = 1116	*n* = 73	*n* = 18	*n* = 1831	*n* = 1469
**L1A**	4.0																
**L1B**	6.9	5.0															
**L1C**	10.1	10.0	7.0														
**L1D**	10.9	10.5	12.6	11.0													
**L1Dalpha**	11.2	10.7	13.1	10.6	10.0												
**L1Dbeta**	9.0	9.6	9.3	11.1	11.7	7.0											
**L1E**	13.4	12.5	13.2	14.2	14.3	13.6	5.0										
**L1F**	9.5	10.1	10.4	12.1	12.5	9.5	14.7	6.0									
**L1G**	8.1	6.7	11.0	12.0	12.2	10.4	13.4	11.0	6.0								
**L1H**	9.0	10.5	9.3	11.5	12.1	7.9	14.7	9.7	11.2	4.0							
**L2**	14.5	14.4	15.4	14.4	14.4	14.5	16.0	14.5	15.7	14.4	13.0						
**L4**	14.6	14.6	17.1	15.1	14.9	15.9	18.9	16.4	15.8	16.5	17.9	16.0					
**L5**	15.1	15.4	17.3	14.2	13.9	16.1	17.7	17.3	16.6	16.2	14.4	15.6	5.0				
**L6**	19.2	18.6	19.4	17.6	17.3	19.4	19.1	20.0	19.9	18.9	16.8	20.1	14.6	7.0			
**L7**	12.5	12.0	14.1	11.4	11.1	12.9	13.7	14.2	13.4	13.5	12.6	13.8	9.6	13.8	5.0		
**L8**	13.4	13.5	14.9	12.5	12.3	14.1	16.4	15.3	15.4	14.0	13.8	15.4	11.9	14.6	9.4	6.0	
**L9**	15.6	15.3	16.5	13.8	13.4	15.9	17.3	16.6	16.7	16.0	14.4	17.0	13.1	15.4	11.1	10.6	10.0
**Prevacent - Elanco (L1)**	11.7	11.9	11.7	12.8	13.4	8.4	14.2	11.0	12.6	9.5	15.7	17.5	17.0	21.9	13.7	15.9	17.7
**Ingelvac PRRSV MLV - BI (L5)**	14.8	15.2	17.3	14.1	13.8	16.0	18.2	17.5	16.4	16.0	14.6	15.7	3.1	14.5	9.3	12.0	13.2
**Prime Pac PRRSV RR - Merck (L7)**	12.6	12.2	14.4	10.9	10.6	12.8	14.4	14.1	13.8	12.9	12.3	13.7	8.8	13.0	3.3	8.6	10.4
**Ingelvac PRRSV ATP - BI (L8)**	12.8	13.1	14.7	11.9	11.6	13.7	15.8	14.8	15.1	13.4	13.3	14.6	10.8	14.1	8.6	4.3	10.0
**Fostera PRRSV - Zoetis (L8)**	14.6	13.9	16.3	12.2	11.8	14.9	17.6	16.0	15.3	15.4	13.8	15.7	10.1	12.8	7.8	7.3	9.1
