# Supplementary figures and images for: Phylogenetic Structure and Sequential Dominance of Sub-Lineages of PRRSV Type-2 Lineage 1 in the United States
Source: Vaccines (Basel). 2021 Jun 5;9(6):608. doi: 10.3390/vaccines9060608 (PMC8229766; doi:10.3390/vaccines9060608)

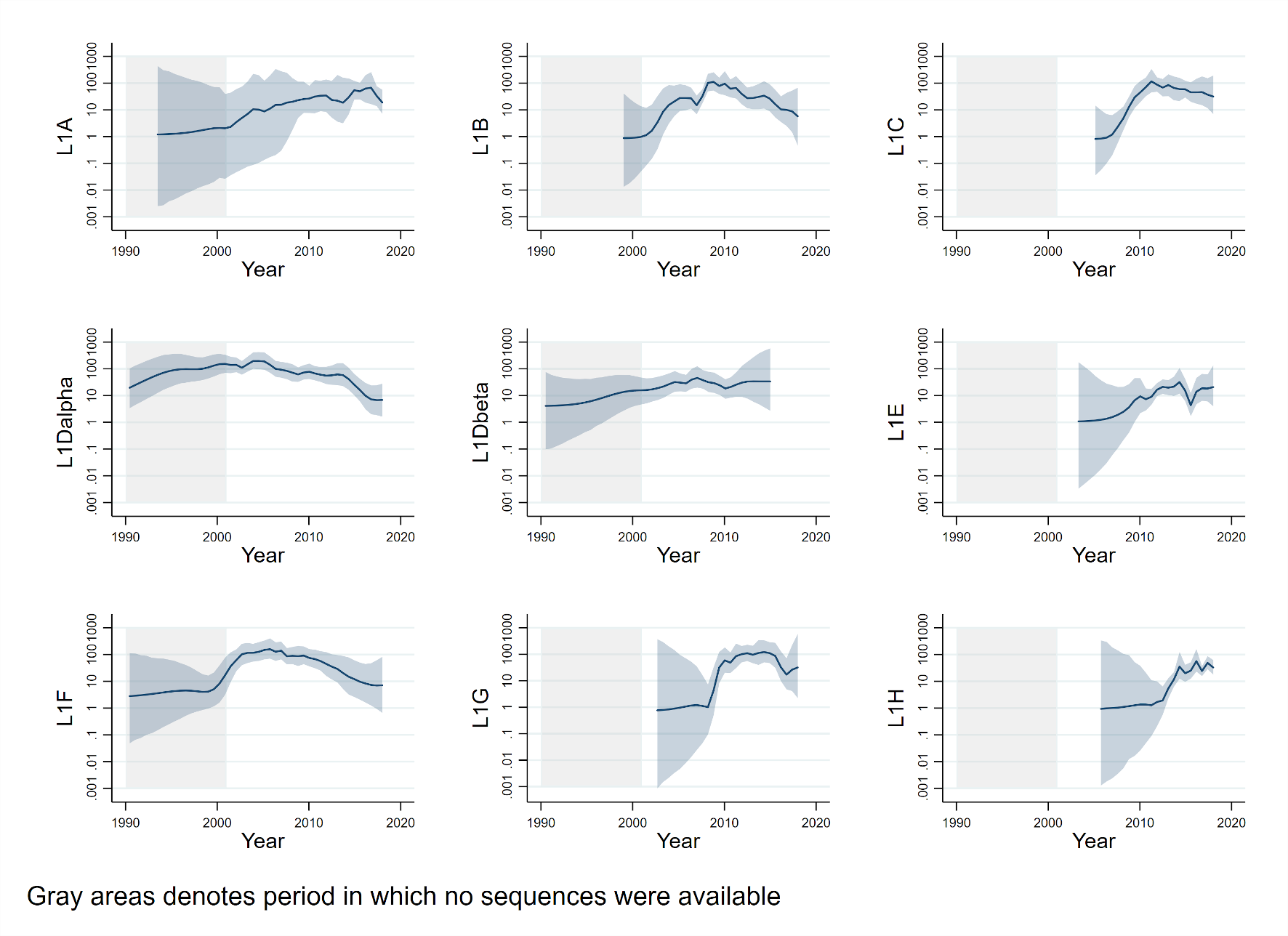

Supplement: Supplementary file 1 [file vaccines-09-00608-s001.zip › Sup Fig 1 BEAST each SL.tif]

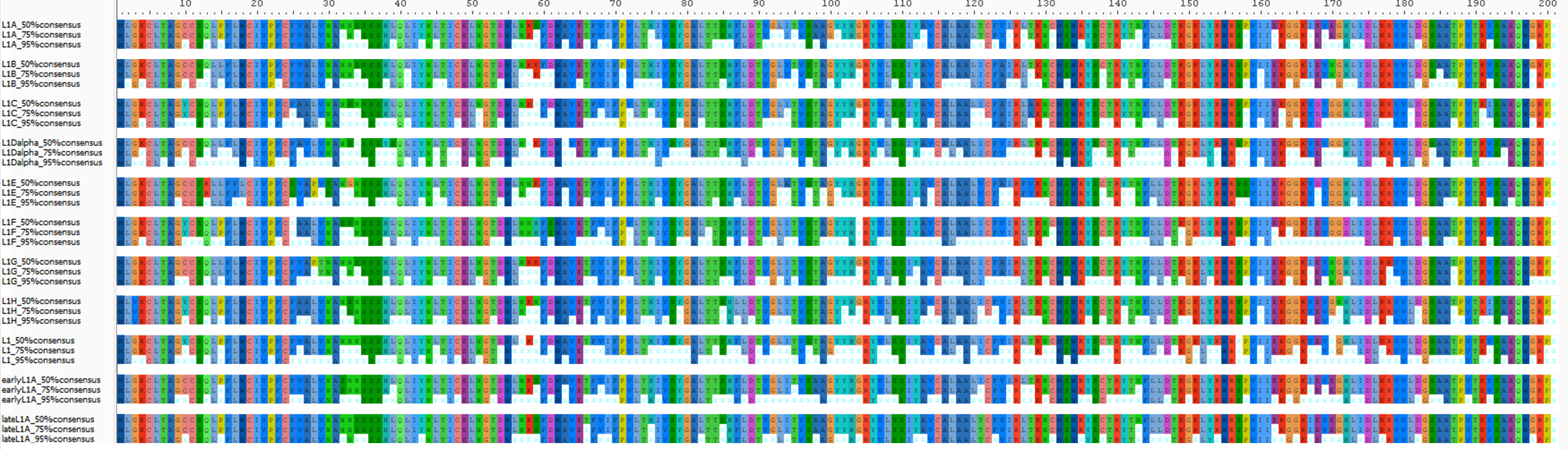

Supplement: Supplementary file 1 [file vaccines-09-00608-s001.zip › Sup Fig 2 50 75 and 95 pc consensus.tif]
